# VEGFR2 targeted antibody fused with MICA stimulates NKG2D mediated immunosurveillance and exhibits potent anti-tumor activity against breast cancer

**DOI:** 10.18632/oncotarget.7501

**Published:** 2016-02-19

**Authors:** Xie Wei, Fang Liu, Youfu Wang, Xueyan Ren, Tong Wang, Zhiguo Chen, Mingying Tang, Fumou Sun, Zhaoting Li, Min Wang, Juan Zhang

**Affiliations:** ^1^ State Key Laboratory of Natural Medicines, School of Life Science & Technology, China Pharmaceutical University, Nanjing, China

**Keywords:** antibody fusion protein, VEGFR2, MICA, anti-angiogenesis, immunosurveillance

## Abstract

Binding of MHC class I-related chain molecules A and B (MICA/B) to the natural killer (NK) cell receptor NK group 2, member D (NKG2D) is thought critical for activating NK-mediated immunosurveillance. Angiogenesis is important for tumor growth and interfering with angiogenesis using the fully human IgG1 anti-VEGFR2 (vascular endothelial growth factor receptor 2) antibody (mAb04) can be effective in treating malignancy. In an effort to make mAb04 more effective we have generated a novel antibody fusion protein (mAb04-MICA) consisting of mAb04 and MICA. We found that mAb04-MICA maintained the anti-angiogenic and antineoplastic activities of mAb04, and also enhanced immunosurveillance activated by the NKG2D pathway. Moreover, in human breast tumor-bearing nude mice, mAb04-MICA demonstrated superior anti-tumor efficacy compared to combination therapy of mAb04 + Docetaxel or Avastin + Docetaxel, highlighting the immunostimulatory effect of MICA. In conclusion, mAb04-MICA provided new inspiration for anti-tumor treatment and had prospects for clinical application.

## INTRODUCTION

Angiogenesis, a critical hallmark of malignancy, is principally driven by interactions between vascular endothelial growth factors (VEGFs) and VEGF receptors (VEGFRs) [1-3]. Growth and metastasis have been strongly linked to angiogenesis in the majority of cancers, including metastatic breast carcinoma [4]. Bevacizumab is the first anti-angiogenic agent to be approved by the US FDA targeting VEGF. Whereas, available clinical data show that blocking VEGF by Bevacizumab does not completely inhibit tumor angiogenesis [5]. Ramucirumab is a fully human monoclonal (IgG1) anti-VEGFR2 antibody approved for clinical treatment of malignancy by US FDA in 2014. However, Phase III trials in patients with human epidermal growth factor receptor-2 (HER2^−^) metastatic breast carcinoma indicated that, overall survival (OS) was not prolonged by the addition of Ramucirumab to the first-line chemotherapeutic drug (Docetaxel). In addition, fatigue, hypertension, febrile neutropenia and palmar-plantar erythrodysesthesia syndrome (PPE syndrome) were common serious side effects in the Ramucirumab treatment group [6-8].

The anti-cancer activity of unmodified antibodies depend in large part on the fragment crystallizable (Fc) region of the antibody, which interacts with Fc gamma receptors (FcγRs) on effector immune cells to stimulate antibody-dependent cellular cytotoxicity (ADCC) [9-11]. However, functional polymorphisms of FcγRIIa and FcγRIIIa in human may lead to different affinities to the Fc domains resulting in varying clinical responses [12, 13].

NK cells, the major effectors of ADCC, constitutively express FcγRIIIa [14] and regulated by several inhibitory or activating receptors [15-18]. In addition to FcγRIIIa, NK cells express a variety of activating receptors, among which NKG2D was shown to play an important role in tumor cell rejection and tumor immunosurveillance [19-21]. In humans, NKG2D binds to MHC class I-related chain (MIC) A, MICB, and UL16-binding proteins (ULBPs) whose expression is restricted or absent on normal tissues, but is induced in situations of stress and disease including cancer [22]. Indeed, MICA, one of the major ligands for NKG2D, is often overexpressed in many tumor tissues from patients with epithelial tumors and some primary leukemia cells [23-26]. However, since the tumors progressed despite the expression of MICA, it appeared that the MICA-NKG2D system was functionality compromised in these particular patients [26-28]. Studies found that tumor cells avoid the response of NKG2D through shedding MICA from the cell surface, and this soluble MICA hinders recognition of the MICA-expressing tumor cells, thereby impairing the anti-tumor immune response. Significantly increased serum levels of soluble MICA were found to correlate with poor clinical outcome in patients suffering from various types of cancer [27-30].

Based on these observations, alternate approaches were sought to express NKG2D-ligands on the tumor cells and thereby trigger an effective immune response by NK cells. In one approach a scFv (single chain fragment variable) targeting carcino embryonie antigen (CEA) was joined to ULBP2 (UL16-binding protein 2) ligand; this fusion protein successfully activated and retargeted NK cells against tumor cell lines or primary patient tumor cells, and showed antitumor activity in both allogeneic and autologous settings [26, 31]. Similarly, the chemical conjugates associating recombinant MICA molecule with antigen-binding fragments (Fab) of different monoclonal anti-tumor antibodies were demonstrated to specifically bind TAAs (tumor associated antigens) and to stimulate NKG2D-dependent lysis of resistant tumor cells by human NK cells [32, 33].

We previously generated a novel human IgG1 antibody (mAb04) specific for VEGFR2. This antibody had high affinity to VEGFR2 and exhibited anti-angiogenic activity both *in vitro* and *in vivo* [34]. To enhance the immunostimulatory activity of mAb04, we have now fused it to MICA. The resulting antibody-based fusion protein (mAb04-MICA) showed therapeutic efficacy in the nude mice transplanted with human breast tumor cells. mAb04-MICA represents a novel recombinant bispecific antibody-ligand construct in which a fully human IgG1 antibody is used to target tumor cells and the associated MICA stimulates cell killing effect of NK cells.

## RESULTS

### Generation and identification of mAb04-MICA

**Figure 1:**
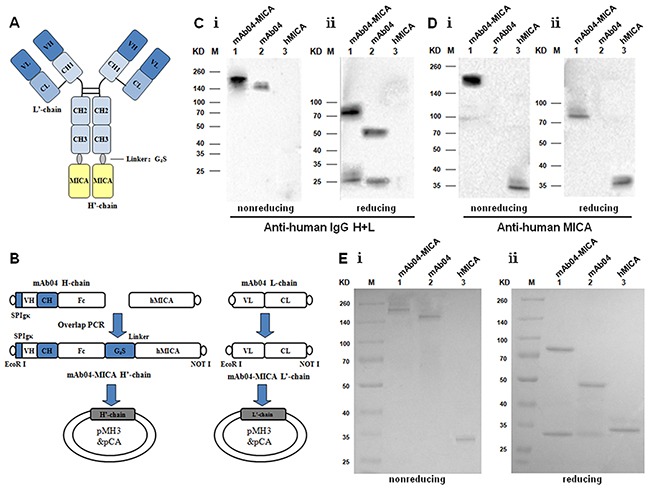
Construction and production of mAb04-MICA fusion protein **A.** Structure diagram of the fusion protein. **B.** Flow diagram for the construction of expression plasmids. **C, D.** Western blot analysis for the assembling of purified mAb04-MICA. C i: anti-human IgG (H+L) antibody under nonreducing condition; C i: anti-human IgG (H+L) antibody under reducing condition. D i: anti-human MICA antibody under nonreducing condition; D i: anti-human MICA antibody under reducing condition. Lane 1: mAb04-MICA; Lane 2: mAb04; Lane 3: hMICA. **E.** SDS-PAGE analysis for the purity of mAb04-MICA. Ei: nonreducing condition; Ei: reducing condition. Lane 1: mAb04-MICA; Lane 2: mAb04; Lane 3: hMICA.

The mAb04-MICA fusion protein was purified as described in Materials and Methods (Figure 1A and 1B). Western blot analysis utilizing anti-human IgG (H+L) (Figure 1C) and anti-human MICA antibody (Figure 1D) indicated that the complete antibody fusion protein (210 KD) contained both mAb04 and hMICA with MICA attached to the H chain. SDS-PAGE and staining with Coomassie Brilliant Blue confirmed the purity of the isolated antibody fusion protein mAb04-MICA (Figure 1E).

### mAb04-MICA bound specifically to KDR3 and NKG2D

**Figure 2:**
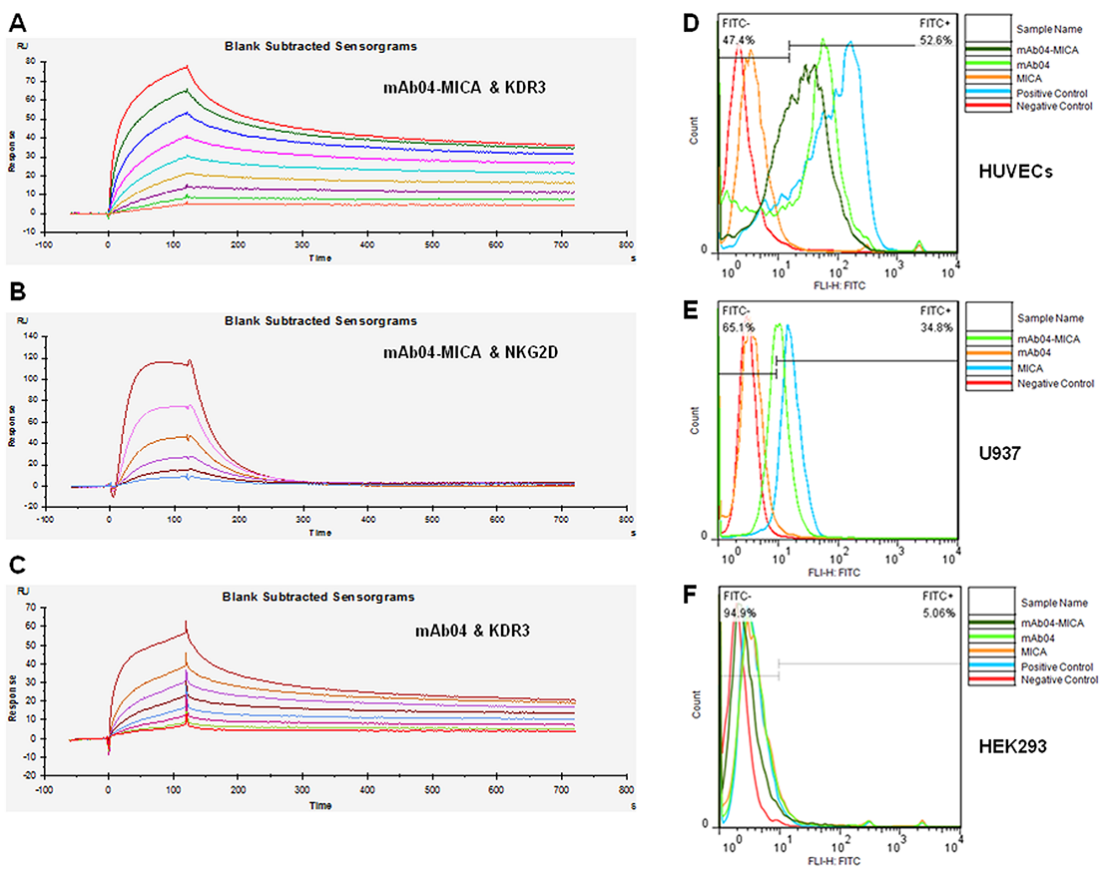
mAb04-MICA bound specifically to KDR3 and NKG2D **A.** Set of sensorgrams of KDR3 binding with mAb04-MICA. The association rate increased with increasing concentration of the KDR3 (from bottom to top), ranging from 0.78125 nM to 200 nM. The complex dissociated when buffer flowed through at 120 s. K_D_ (M): 1.29×10^−9^. **B.** Set of sensorgrams of NKG2D binding with mAb04-MICA. The concentration of NKG2D (from bottom to top), ranged from 7.8125 nM to 250 nM. K_D_ (M): 7.102×10^−7^. **C.** Set of sensorgrams of KDR3 binding with mAb04. The concentration of KDR3 (from bottom to top), ranged from 0.625 nM to 160 nM. K_D_ (M): 1.05×10^−9^. **D.** mAb04-MICA and mAb04 showed high affinity with VEGFR2 over-expressing HUVECs, the binding rate was 52.6% and 51% respectively. **E.** mAb04-MICA could bind to NKG2D over-expressing U937 cells (34.8%). The binding rate was relatively lower than hMICA (63.4%). **F.** The VEGFR2/NKG2D-negative cell line HEK293 was employed as a negative control, data of this group demonstrated the specificity of binding.

The binding of KDR3 and NKG2D to immobilized mAb04-MICA was evaluated, and the 2:1 binding model was used for affinity and kinetic analysis. mAb04-MICA exhibited high affinity to KDR3 (*k_a_* (1/Ms): 6.18×10^5^, *k_d_* (1/s): 8.00×10^−4^, K_D_ (M): 1.29×10^−9^) (Figure 2A), similar to that of mAb04 (*k_a_*: 9.83×10^5^, *k_d_*: 1.03×10^−3^, K_D_: 1.05×10^−9^) (Figure 2C). The affinity constant between mAb04-MICA and NKG2D (*k_a_*: 2.65×10^8^, *k_d_* (1/s): 188.2, K_D_ (M): 7.102×10^−7^ (Figure 2B)) was slightly lower than that of MICA (K_D_: 3.95×10^−8^) [36]. Above, the immobilized mAb04-MICA demonstrated specificity and affinity to soluble KDR3 and NKG2D, confirming that mAb04-MICA retained binding capacity of each portion *in vitro*.

Specific binding of mAb04-MICA to HUVECs (52.6%) and NKG2D over-expressing U937 cells (34.8%) was observed (Figure 2D and 2E) whereas nonbinding with HEK293 (Figure 2F). Thus, mAb04-MICA could recognize both VEGFR2 over-expressing and NKG2D over-expressing cells.

### mAb04-MICA inhibited HUVECs proliferation, invasion and tube formation

**Figure 3:**
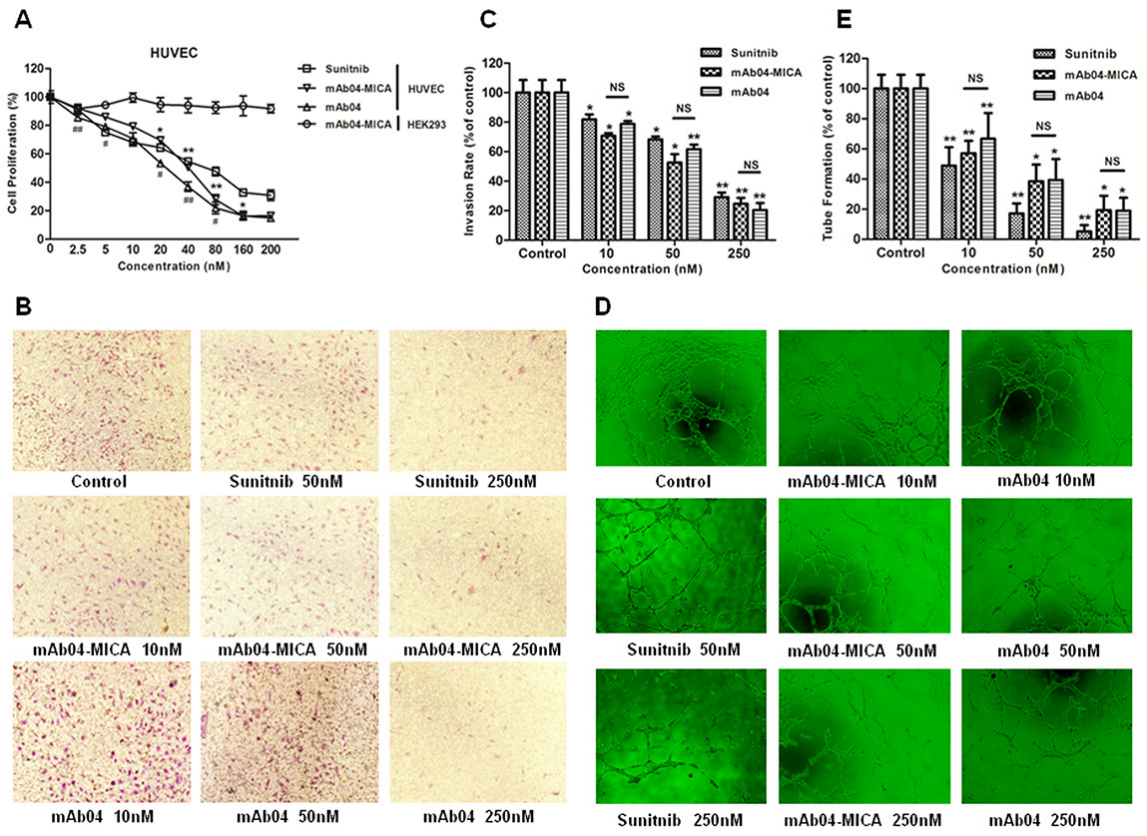
mAb04-MICA inhibited the proliferation, invasion and tube formation of HUVECs **A.** mAb04-MICA (Data were presented as the mean ± SD, n = 5, *p < 0.05, **p < 0.01)/mAb04 (n = 5, ^#^p < 0.05, ^##^p < 0.01) inhibited the proliferation of HUVECs with VEGF stimulated in dose-dependent manner. **B, C.** Photomicrographs and quantative analysis of transwell invasion assay indicated that mAb-04 suppressed the invasion of HUVECs in dose-dependent manner. **D, E.** HUVECs tube-like photomicrographs and quantitative analysis demonstrated the significant effects of mAb04-MICA/mAb04 on HUVECs tube formation (Data were presented as the mean ± SD, n = 5 (C, E), *p < 0.05, **p < 0.01, NS, no significance).

The inhibition of mAb04-MICA on HUVECs was in a dose-dependent manner, with inhibitory rate (IC_50_ = 23.876 ± 1.378 nM) close to that of mAb04 but higher than that of Sunitnib. However, no inhibition on HEK293 was seen (Figure 3A), indicating the specificity of mAb04-MICA to HUVECs. Transwell invasion assay (Figure 3B and 3C) showed that the number of invasive cells in the lower chamber was significantly reduced with increasing concentrations of the treatments. The maturation of endothelial cells into a capillary tube, a critical early step of angiogenesis, was gradually abrogated with increasing concentrations of the treatments (Figure 3D and 3E). In conclusion, no significant difference was seen between mAb04 and mAb04-MICA in terms of the anti-angiogenic activities, with similar effect to Sunitnib, a well defined tyrosine kinase inhibitor.

### mAb04-MICA inhibited proliferation, promoted apoptosis and altered cell cycle of breast cancer cells

**Figure 4:**
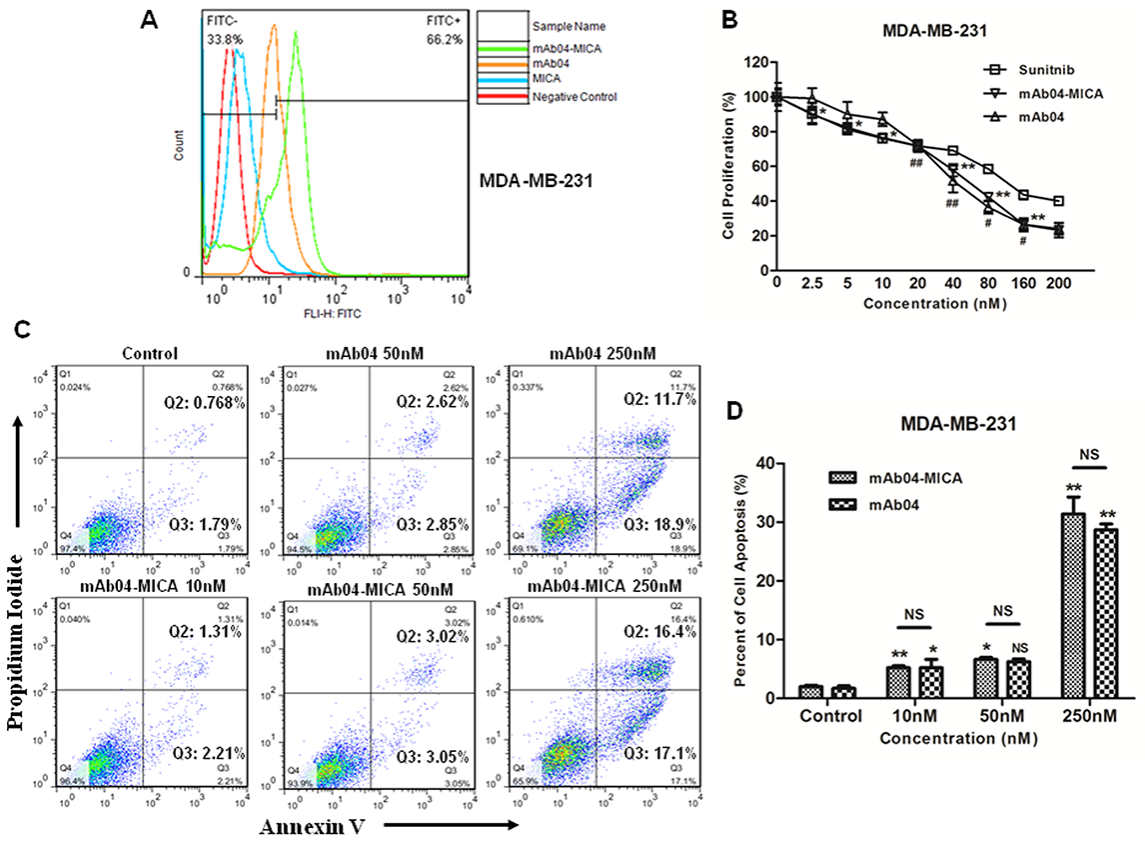
mAb04-MICA inhibited proliferation and induced apoptosis of MDA-MB-231 cells **A.** mAb04-MICA/mAb04 exhibited high binding rate with MDA-MB-231 cells, reaching 66.2% and 50.3% respectively. **B.** mAb04-MICA (Data were presented as the mean ± SD, n = 5, *p < 0.05, **p < 0.01)/mAb04 (n = 5, ^#^p < 0.05, ^##^p < 0.01) inhibited the proliferation of MDA-MB-231 cells with the IC_50_ (53.196 ± 1.726 nM), similar to that of mAb04 (57.272 ± 1.758) and Sunitnib (73.392 ± 0.952). **C.** MDA-MB-231 cells were incubated with various treatments at 37°C for 48 h and analyzed by flow cytometry following staining with Annexin V-FITC and PI. The percentage of cells in each quadrant was indicated. **D.** Quantitative analysis of apoptosis assay (Data were presented as the mean ± SD, n = 3, *p < 0.05, **p < 0.01).

**Figure 5:**
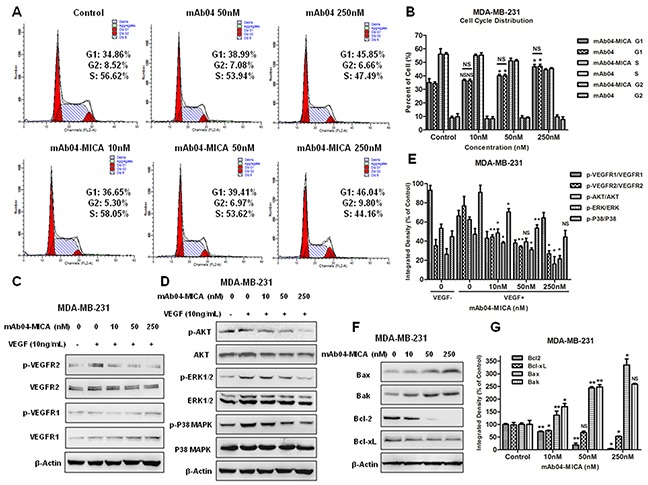
mAb04-MICA induced the G0/1 phase arrest in MDA-MB-231 cells, inhibited VEGFR2 pathway and modulated the Bcl-2 family protein **A.** Cell cycle analysis of MDA-MB-231 cells that were incubated with various treatments for 48 h and stained with PI. The percentage of cells in each phase was indicated. **B.** Quantitative analysis of cell cycle assay. **C, D.** Western blot analysis for p-VEGFR2/VEGFR2, p-AKT/AKT, p-ERK/ERK and p-P38/P38 of MDA-MB-231 cells treated with mAb04-MICA. Equal loading of protein was confirmed by stripping the immunoblot and reprobing it for β-actin. **E.** Gray scanning and data statistics of C, D. **F.** Western blot analysis for Bcl-2 family protein of MDA-MB-231 cells treated with mAb04-MICA. **G.** Gray scanning and data statistics of (F) Data were presented as the mean ± SD, n = 3 (B, E, G), *p < 0.05, **p < 0.01.

mAb04-MICA exhibited a relatively higher binding rate with MDA-MB-231 cells (Figure 4A) and significantly inhibited the proliferation of MDA-MB-231 cells (Figure 4B). To determine if the observed inhibition of proliferation following treatment with mAb04-MICA was associated with apoptosis and alterations in cell cycle, MDA-MB-231 cells were treated with various concentrations of mAb04-MICA for 48 h and examined by flow cytometry. Similar dose-dependent increase in apoptosis was seen following treatment with mAb04-MICA and mAb04 (Figure 4C and 4D). Cell cycle analysis of both mAb04-MICA or mAb04 treated MDA-MB-231 cells revealed that a substantial proportion was arrested at the G0/1 checkpoint (Figure 5A and 5B).

### mAb04-MICA down regulated VEGFR2 pathway and modulated the Bcl-2 family protein

To investigate the antineoplastic mechanism of mAb04-MICA on MDA-MB-231 cells, the phosphorylation of VEGFR2 and downstream signaling proteins were examined. mAb04-MICA dose-dependently inhibited VEGF induced phosphorylation of VEGFR2 (Figure 5C and 5D) and AKT/ERK/P38 MAPK (Figure 5E). Examination of the Bcl-2 family proteins showed that mAb04-MICA treatment greatly reduced the expression of anti-apoptotic protein Bcl-2 and Bcl-xL, also increased the expression of pro-apoptotic proteins Bak and Bax (Figure 5F and 5G).

### mAb04-MICA enhanced PBMCs or NK92-FcR cells cytotoxicity and maintained CDC

**Figure 6:**
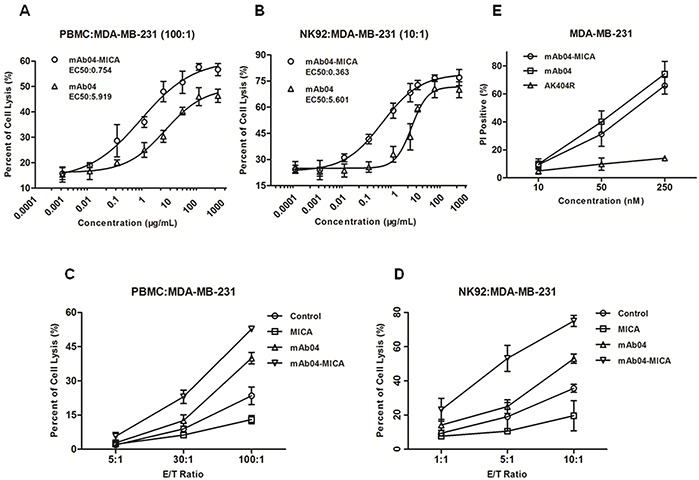
mAb04-MICA enhanced the cytotoxic effect of PBMC/NK92 cells compared to mAb04, and maintained the CDC effect **A, B.** Cytotoxicity assay to assess the PBMC/NK92 cell-mediated killing of MDA-MB-231 cells. The EC_50_ of mAb04-MICA/mAb04 were fitted. **C, D.** MDA-MB-231 cells were used as target cells and PBMC/NK92 cells as effect cells for a LDH release assay. The increase of cell lysis was enhanced significantly upon treatment with mAb04-MICA compared to mAb04. Measurements were performed in triplicates (A-D). **E.** MDA-MB-231 cells were incubated in triplicate with mAb04-MICA/mAb04/AK404R and rabbit serum. Following washing, cells were stained with PI and analyzed by flow cytometry to assess viability. Data were expressed as the mean ± SD of PI-positive percentage.

Although both mAb04-MICA and mAb04 increased cellular cytotoxicity in a dose-dependent manner, compared to mAb04, mAb04-MICA triggered stronger cellular cytotoxicity with a relatively lower EC_50_ (Figure 6A and 6B). Assessing immune cell-mediated killing at different effector-target (E:T) ratios also showed mAb04-MICA exerted stronger cytotoxicity than mAb04 in an E:T ratio-dependent manner (Figure 6C and 6D). The specific lysis of MDA-MB-231 was reduced after treatment with free soluble MICA, indicating that free soluble MICA weakened the PBMCs or NK92-FcR cells cytotoxicity [41, 42]. Analogous observations were seen when MDA-MB-435 cells (Supplementary Figure S1A and S1B) were involved as the target cells (Supplementary Figure S1C and S1D). However, the lymphocytes mediated inferior cytotoxicity to MDA-MB-435 cells, which was in accordance with flow cytometry data (Figure 4A and Supplementary Figure S1A), suggesting the cytotoxicity effect was consistent with the recognition of fusion protein to the target cells, the higher binding rate, the stronger cytotoxicity. mAb04-MICA and mAb04 were found similar in inducing complement-dependent cytotoxicity (Figure 6E). While, AK404R (the variable fragment of mAb04) lacking Fc fragment did not exhibit CDC activity.

### mAb04-MICA increased NK92 cells degranulation and cytokine production

**Figure 7:**
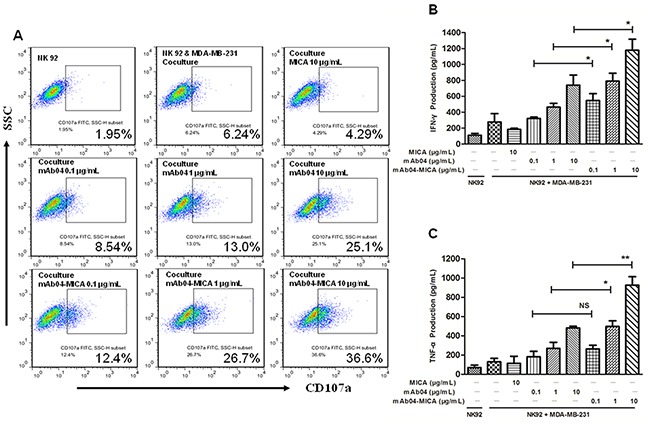
Degranulation of NK92 and the expression of cytokines were up regulated in mAb04-MICA group compared to mAb04 **A.** Flow cytometry analysis of CD107a expression on IL-2 activated NK92 cells after exposure to MDA-MB-231 cells for 4 h in the presence of the treatments. The E/T ratio was 10:1. **B, C.** ELISA detected the IFNγ and TNF-α concentrations after NK92 cells co-cultured with MDA-MB-231 cells for 4 h at E:T ratio (10:1). These results obtained on triplicate samples were presented as the mean ± SD, *p < 0.05, **p < 0.01.

**Figure 8:**
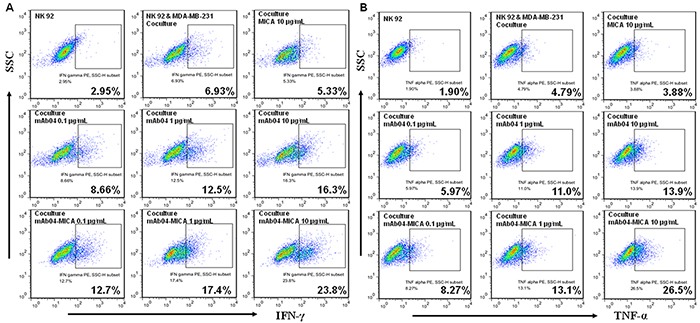
NK92 cells secreted more cytokines when treated with mAb04-MICA in the coculture with MDA-MB-231 cells **A, B.** Flow cytometry data represented the distribution of cytokine positive cells among NK92 cells, which indicated the proportion of NK92 cells expressing IFNγ/TNF-α along the x-axis increased as the treatments concentration increased. The percentage of IFNγ/TNF-α positive cells was calculated by FlowJo software.

To investigate how mAb04-MICA treatment modified the NKG2D-mediated killing, we assessed degranulation of NK92 cells using CD107a as a marker [43]. The CD107a positive rate of NK92 cells was 26.7% and 36.6% following treatment with 1 and 10 μg/mL mAb04-MICA respectively, higher than the 13.0% and 25.1% rate following treatment with mAb04 at the same concentration (Figure 7A). Similarly, mAb04-MICA treatment could increase NK92 cells degranulation when co-cultured with MDA-MB-435 cells (Supplementary Figure S2A). A slight variation on this was that the activation extent of NK92 cells was lower when MDA-MB-435 cells acted as the target cells; most tellingly, the CD107a positive rate was 24.4% which was 12.2% lower than that of MDA-MB-231 cells. ELISA assay revealed IFNγ and TNF-α production by NK92 cells was enhanced upon exposure to mAb04-MICA/mAb04, and in which mAb04-MICA was more effective (Figure 7B and 7C). Analysis by *t* test showed a significant difference of secretory cytokine production between mAb04-MICA and mAb04 group at the same concentration. FACS analysis reconfirmed that NK92 cells treated with mAb04-MICA had higher expression of IFNγ and TNF-α than those treated with mAb04 (Figure 8A and 8B). It is noteworthy that ELISA assay (Supplementary Figure S2B and 2C) and FACS analysis (Supplementary Figure S3A and 3B) showed the similar immunomodulatory effects of mAb04-MICA on MDA-MB-435 cells, and the effect intensity was correlated with the binding rate of mAb04-MICA to VEGFR2-expressed cancer cells.

### mAb04-MICA inhibited tumorigenicity of breast cancer xenografts

**Figure 9:**
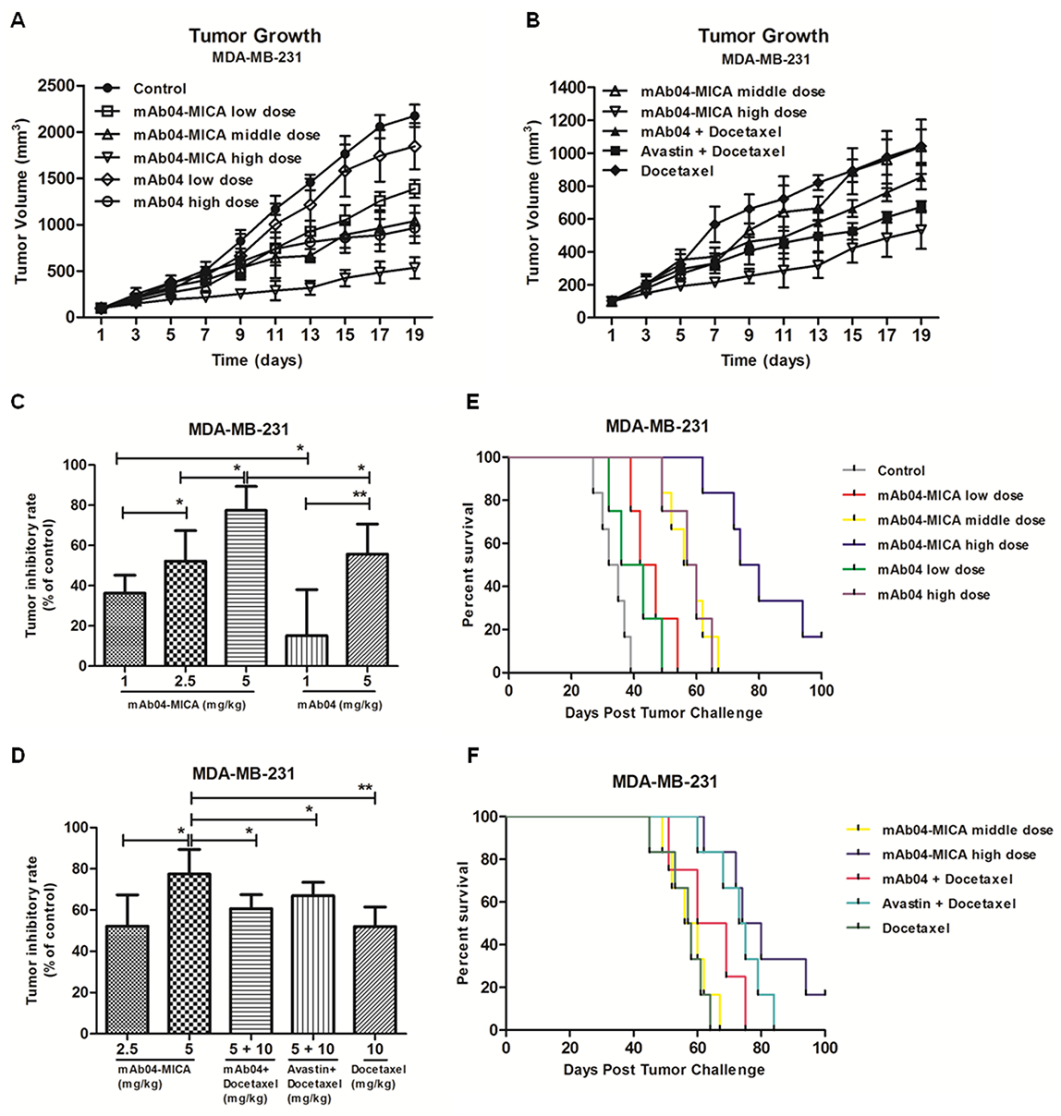
mAb04-MICA demonstrated *in vivo* efficacy against a MDA-MB-231 xenograft **A, B.** Tumor growth curves for nude mice. Each BALB/c nude mouse was subcutaneously injected 1×10^7^ MDA-MB-231 cells for different treatment. Treatment began following tumor development, and the measurement of tumor volume started as well. **C, D.** Tumor inhibition rates of different dosage groups. mAb04-MICA significantly improved tumor inhibition rate compared to mAb04/Docetaxel. Data were presented as the mean ± SD, *p < 0.05, **p < 0.01. **E, F.** Survival curves for nude mice bearing tumor. mAb04-MICA had noteworthy survival benefit compared to mAb04 + Docetaxel or Avastin + Docetaxel.

Treatment of MDA-MB-231 xenografted nude mice with mAb04-MICA was more effective than that with mAb04 in inhibiting tumor growth, achieving 36.28% and 77.43% tumor growth inhibition at doses of 1 and 5 mg/kg compared to 15.13% and 55.71% for mAb04, respectively. In addition, high dose treatment of mAb04-MICA was superior to the combination therapy groups (60.73%, mAb04 + Docetaxel, 66.99%, Avastin + Docetaxel) (Figure 9A to 9D). Consistent inhibition was observed in MDA-MB-435 xenografts (Supplementary Figure S4A to S4D).

Treatment with mAb04-MICA also prolonged survival. All mice bearing MDA-MB-231 xenograft treated with PBS succumbed to tumor at day 39 (Figure 9E). In this setting, mAb04-MICA at a dose of 5 mg/kg increased median survival by 44 days, comparably mAb04 25 days (Figure 9F), mAb04 + Docetaxel 31 days and Avastin + Docetaxel 34 days, respectively. In terms of MDA-MB-435 tumor-bearing mice, treatment with mAb04-MICA significantly prolonged the survival compared to the control group (Supplementary Figure S4E and S4F).

### mAb04-MICA inhibited markers of proliferation and angiogenesis in tumor xenograft

**Figure 10:**
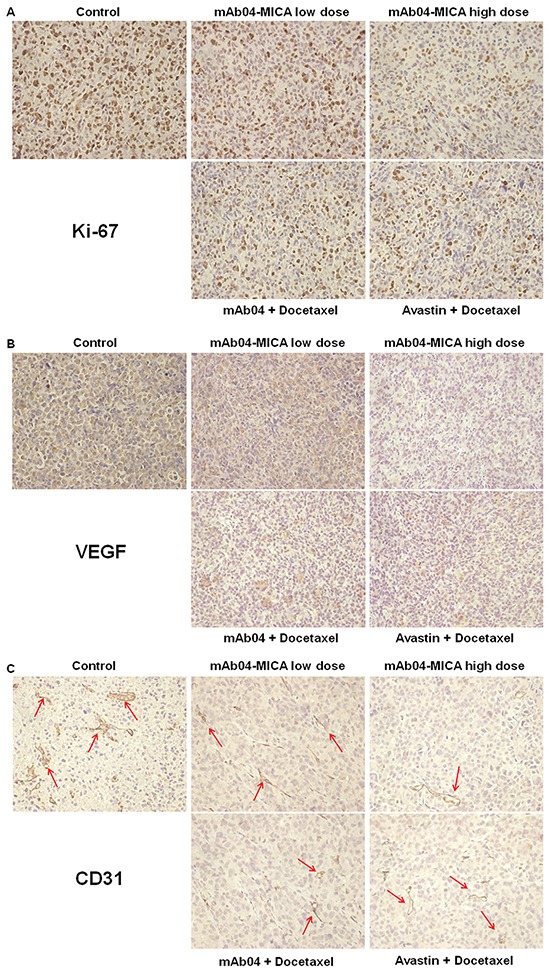
mAb04-MICA reduced markers of proliferation and angiogenesis in MDA-MD-231 xenograft **A.** IHC staining of Ki-67 on paraffin sections of xenografted tumor. Ki-67^+^ cells were identified with an anti-Ki-67 antibody (brown staining). **B.** IHC staining of VEGF (brown staining). **C.** IHC staining of CD31. The CD31^+^ blood vessels were identified with an anti-CD31 antibody (brown staining, indicated by the red arrows). Photomicrographs showed representative pictures from 3 independent tumor samples.

IHC demonstrated that there was a significant decrease in the numbers and intensity of cell proliferation marker Ki-67 in mAb04-MICA treated tumors compared to untreated groups, with a slight decrease compared to mAb04 + Docetaxel or Avastin + Docetaxel treated group (Figure 10A).

Tumor sections stained with anti-VEGF (Figure 10B) and anti-CD31 (Figure 10C) antibodies showed reduced intensity of staining in the mAb04-MICA treated groups. The density of tumor neovascularization was lower in the mAb04-MICA (5 mg/kg) treated group than mAb04 + Docetaxel or Avastin + Docetaxel group. The enhanced inhibition of Ki-67/VEGF/CD31 by mAb04-MICA were consistent with increased anti-tumor effects resulting from the presence of MICA.

### mAb04-MICA increased tumor-infiltrated NK cells and stimulated the expression of IFNγ and TNF-α

**Figure 11:**
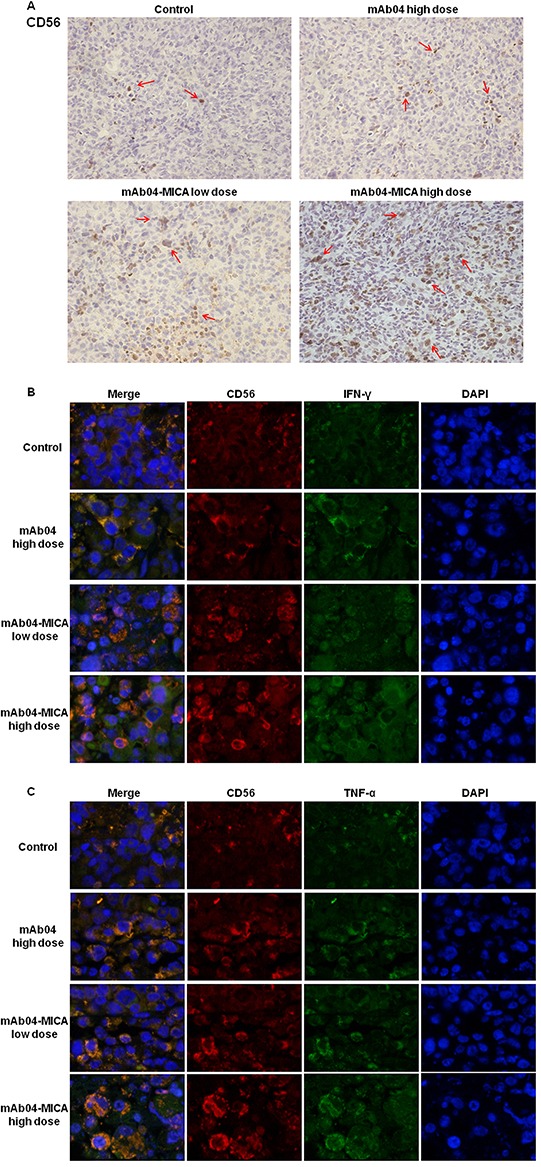
mAb04-MICA aggravated soakage of NK cells in MDA-MB-231 tumor tissue and increased the production of IFNγ and TNF-α by NK cells **A.** Infiltrated CD56^+^ cells were detected by IHC staining (brown staining, indicated by the red arrows) on serial sections, demonstrating more distribution of NK cells with mAb04-MICA treatment. **B, C.** IF double staining of CD56 (red fluorescence) and IFNγ/TNF-α (green fluorescence) to determine the expression level of IFNγ/TNF-α by NK cells. The orange staining cells after merged indicated the IFNγ/TNF-α expressing NK cells, which increased with the increase of dose.

IHC analysis (Figure 11A/Supplementary Figure S5A) revealed the level of infiltrating CD56^+^ cells (CD56 is not strictly specific for NK cells, but we estimated that CD56 gave a reasonable representation of NK cells in the tumor tissue [39]) was considerably higher in tumor tissues of mice treated with mAb04-MICA than tumors from mice treated with the same dose of mAb04. Significantly higher levels of secretion and expression of IFNγ and TNF-α were also found in the mAb04-MICA treated group (Figure 11B and 11C/Supplementary Figure S5B and S5C). The infiltrated NK cells and the secreted immune cytokines in MDA-MB-435 were less than those in MDA-MB-231 with the same treatments, which was consistent with the cytotoxicity assay and immunoregulatory experiments *in vitro*. It was therefore mAb04-MICA enhanced immunosurveillance mediated by NK cells against VEGFR2 positive breast cancer cells, and the activities of immunosurveillance and inhibiting tumor were proportional to the binding of mAb04-MICA to cancer cell surface.

## DISCUSSION

Disseminating tumors including breast cancer remain a leading cause of death in industrialized countries [44]. HER2^+^ and ER^+^ tumors are candidate for anti-HER2 therapy and hormonotherapy, respectively. The basal-like phenotype represents only 10 to 15% of mammary carcinomas [45, 46] corresponding to TN tumors, which express neither HER2 nor ER. The TN tumors are characterized by a high proliferation index and require efficient neoangiogenesis, which suggests that targeting VEGF-VEGFR2 signal pathway may be an effective treatment strategy [47, 48]. The phase III clinical trials showed that HER2^−^ tumors, are likely to benefit from bevacizumab therapy in combination with Taxane, but there were many serious side effects [47]. Although the progression-free survival and overall survival of Ramucirumab + Taxol group trended to be prolonged compared to the control group in the phase III clinical trials of HER2^−^ advanced breast cancer, but it was disappointing that there was no statistical significance [8]. Hence, new clinical therapeutic approaches for the treatment of HER2^−^ breast carcinoma are urgently needed.

mAb04-MICA was designed and produced with the goal of reinforcing the immune surveillance activity of NK cells while retaining the anti-angiogenic and antineoplastic activity of mAb04. mAb04-MICA localized in tumor lesions via the recognition of mAb04 to tumor cell surface VEGFR2, and attracted NK cells to the tumor lesions through the associated MICA. Further, the infiltrated NK cells were triggered by the immunoligands to lyse tumor cells. This immunotherapeutic tool represents a novel strategy to trigger the NK cell-based specific immune response. As hypothesized, tumor cells, which are otherwise resistant, can be rendered susceptible to NK cells [49-52].

Both p-VEGFR1 and VEGFR1 were found unexpectedly up regulated in response to high concentrations of mAb04-MICA. We speculated that the interaction of VEGF with VEGFR2 was blocked by mAb04-MICA, and presumably that the excessive VEGF bound with VEGFR1 and stimulated the phosphorylation. Nevertheless, the upregulation of p-VEGFR1 and VEGFR1 did not affect the antineoplastic activity of mAb04-MICA. On the one hand, percentages of p-VEGFR1/VEGFR1 were nearly consistent. On the other hand, it is possible that the stimulation of NK cell mediated cytotoxicity was predominant in the anti-tumor efficacy despite of the possible pathological vascularization caused by the increase of p-VEGFR1 and VEGFR1 [53].

The enhanced immunological effect of mAb04-MICA on triggering NKG2D pathway compared to mAb04 was validated with both PBMCs of healthy donors and NK92 cells. The binding rate of mAb04-MICA to NK92 cells significantly decreased after NKG2D targeted RNA silencing (Supplementary Figure S6A and S6B); Supplementary Figure S6C showed that the cytotoxicity mediated by mAb04-MICA reduced obviously when RNA silencing NKG2D on NK92 cells, but maintained similarity to that of mAb04, which indicated that the interaction between MICA and NKG2D was critical for the recognition of mAb04-MICA to NK92 cells and the ADCC function of Fc was not affected.

*In vivo* test showed that mAb04-MICA exhibited remarkable anti-tumor effect and significantly prolonged the survival of tumor-bearing nude mice. We have designed experiments to demonstrate mice have similar NK cells with human directly binding to MICA, which can help explain the anti-tumor effects of mAb04-MICA in the xenograft mouse tumor model (Supplementary Figure S7). Efficient NK cells infiltration into the core of solid tumors is a prerequisite for the success of such immunoligands. While, the infiltration of NK cells in solid tumors is often inefficient [54-57]. IHC analysis confirmed that mAb04-MICA could specifically inhibit the proliferation of tumor cells, down regulate the angiogenesis in tumor tissue and increase the accumulation of NK cells. In addition, the results of IF implied mAb04-MICA significantly increased the secretion of IFNγ and TNF-α by NK cells compared to mAb04. From the above, the synergistic effect of anti-angiogenesis, inhibiting tumor cell proliferation and NKG2D-mediated immunosurveillance resulted in a superior anti-tumor effect *in vivo* to combination therapy of mAb04 + Docetaxel or Avastin + Docetaxel.

In summary, the fusion antibody we designed retained the high affinity, selectively binding property, anti-angiogenic and antineoplastic activity of mAb04 in combination with the NK-activating properties of MICA. Our study clearly indicates that mAb04-MICA is capable of reinforcing NK cell mediated antitumor activity in VEGFR2-expressed breast cancer both *in vivo* and *in vitro*. Thus, mAb04-MICA is a promising new approach for NK cell-based immunotherapy for malignancy and the strategy of treatment with MICA fused antibody can be further applied to other tumor specific markers such as Her2 and EGFR.

## MATERIALS AND METHODS

### Cell lines and primary cells cultures

The Chinese hamster ovary cell line CHO-s (Amprotein Co., Ltd, Hangzhou, China) was maintained in DMEM/F12 medium (Gibco, Grand Island, USA), supplemented with 10% (v/v) fetal bovine serum (FBS, Gibco, Auckland, NZ). Human umbilical vascular endothelial cells (HUVECs) purchased from ScienCell Research Laboratories were cultured in endothelial culture medium (ECM) supplemented with 5% (v/v) FBS and 1% (v/v) endothelial cell growth supplement (ECGS, ScienCell, San Diego, CA). Human embryonic kidney cell line HEK293 preserved in our lab was cultured in DMEM medium (high glucose), supplemented with 10% (v/v) FBS. Human leukemic monocyte lymphoma cell line U937 and the human breast cancer cell lines MDA-MB-231/MDA-MB-435 were purchased from the American type culture collection (ATCC), and maintained in RPMI 1640 and L-15/DMEM medium supplemented with 10% FBS respectively. The natural killer cell line NK92 (presented by Institute of Biophysics, Chinese Academy of Sciences) was modified by the insertion of the genes for human FcγRIIIa-F158 (Genbank ID: NM 001127595); FcγRIIIa transfected NK92 cell (NK92-FcR cell) was cultured in supplemented MEM Alpha Medium, 12.5% (v/v) FBS (Hyclone, Mordlalloc, Aus), 12.5% (v/v) horse serum (Hyclone, Logan, USA), 0.1 mM 2-Mercaptoethanol, 0.2 mM myo-inositol (Sigma-Aldrich, St. Louis/MO, USA), 0.02 mM Folic Acid (Sigma-Aldrich), 200 U/mL hIL-2 (Millipore, Temecula, CA). Peripheral blood mononuclear cells (PBMCs) were obtained from leukapheresis products of healthy individuals by Ficoll-Hypaque density gradient centrifugation.

### Construction and expression of mAb04-MICA fusion protein

The heavy chain (H-chain) gene was prepared according to the DNA sequence of mAb04 (5′-CCCCCAAGCTGGAATTCCACCATGGAGACAGACACACTCCTGC-3′ and 5′-AGGCTGTGGGGCTCACTTCCACCACCTCCTTTACCCGGAGACA-3′), a fully human IgG1 antibody targeting VEGFR2 we reported [34]. The DNA sequence of Homo MICA extracellular domains (1-3) (hMICA) was synthesized after an optimization with CHO-biased codons (Genbank, NM_001289153.1). The amplified hMICA (5′-TCCCTGTCTCCGGGTAAAGGAGGTGGTGGAAGTGAGCCCCACA-3′ and 5′- GCGGCCGCTTACTACCAGTGGCTCTGCAGCACCAG-3′) was linked to the 3′ end of H-chain (5′-CCCCCAAGCTGGAATTCCACCATGGAGACAGACACACTCCTGC-3′ and 5′-GCGGCCGCTTACTACCAGTGGCTCTGCAGCACCAG-3′) with a flexible pentapeptide (Gly-Gly-Gly-Gly-Ser) by overlap PCR, and the newly acquired fusion gene was named H'-chain. The light chain of mAb04-MICA (L'-chain) retained the same gene as that of mAb04 (L-chain). After digestion with *Not|* and *EcoR|*, H'-chain and L'-chain were ligated with pMH3/pCApuro fragments separately, further confirmed by DNA sequencing.

The expression and assembling of the fusion protein were identified through transient transfection. Subsequently, the recombinant vectors (pMH3-H', pMH3-L, pCApuro-H' and pCApuro-L) were introduced into CHO-s cell line with equal moles by electroporation. The high-yield stable clones were selected using 1 mg/mL G418 (Sigma-Aldrich) by Dot blot (HRP conjugated goat-anti-human IgG Fc, Sino Biological Inc., China) and Western blot assay (HRP conjugated goat-anti-human IgG H+L, Sino Biological Inc., China). The fusion protein was purified using protein A affinity chromatography (GE Healthcare, Uppsala, Sweden) and determined by reducing and nonreducing SDS-PAGE (10%).

### Binding affinity and kinetic analysis

The binding kinetics of mAb04-MICA to KDR3 (the extracellular domain 3 of human VEGFR2) and NKG2D were measured with Biacore system (Biacore X100, GE Healthcare, Sweden). Firstly, Anti-Human IgG (Fc) antibody was immobilized on a sensor chip CM5 using Human Antibody Capture Kit (GE Healthcare, Sweden). After mAb04-MICA was captured, soluble recombinant KDR3/NKG2D kept in our lab [35, 36] was injected at different concentrations into running buffer (HBS-EP, pH 7.4). One flow cell of the sensor chip was set as a control. The captured antibody-analyte complex were removed by regeneration solution (3 M magnesium chloride). The association rate constant *k_a_* and dissociation rate constant *k_d_* were calculated and analyzed using the bivalent analyte model and the equilibrium dissociation constant (K_D_) was calculated (K_D_=*k_d_*/*k_a_*).

### Flow cytometry

The binding capability of the fusion protein to native membrane antigen/ligand was detected by flow cytometry assay (FACSCalibur, BD Biosciences, USA). Basically, VEGFR2/NKG2D over-expressing cell lines were treated with 250 nM of mAb04 or mAb04-MICA, followed by species-specific (1:100) fluorescein isothiocyanate (FITC) conjugates.

### Cell proliferation assay

Cells were plated on a 96-well plate with 4×10^3^/well. After an overnight starvation, the cells were treated with different concentrations of the treatments for 1 h at 37°C, followed by the addition of VEGF_165_ at a final concentration of 10 ng/mL. After incubation for 72 h, cell viability was quantified by MTT assay and the inhibitory rates were expressed as percentages of the vehicle control (antibody untreated but induced with VEGF). The IC_50_ values were then calculated by curve fitting.

### Transwell invasion assay

1×10^4^ of HUVECs were suspended in serum-free medium with the addition of various concentrations of the treatments and then plated into the upper wells of 24-well transwell chambers (Millipore, Billerica, USA), which were pretreated with matrigel (BD Biosciences, Bedford, USA). The lower chambers were filled with 600 μL of ECM containing 5% (v/v) FBS and 1% (v/v) ECGS. After a 12-h incubation, non-invasive cells on the upper were removed while the invaded cells were fixed with 4% polyoxymethylene for 20 min and further stained with 1% (w/v) crystalline violet. Images were taken using an OLYMPUS inverted microscope at 100× magnification. The invaded cells were counted from 10 random fields and plotted using Image-pro-plus program and invasion percentages quantified on the basis of untreated group.

### Tube formation assay

2×10^4^ of HUVECs were incubated with different concentrations of the treatments at 100 μL of ECM supplemented with 2% (v/v) FBS, 1% (v/v) ECGS in a matrigel pretreated 96-well plate. After an 8-h incubation, endothelial tubes were photographed using an inverted OLYMPUS microscope at 100× magnification. Subsequently, the endothelial tubes were counted with image-pro-plus program.

### Apoptosis assay

MDA-MB-231 cells were incubated with various treatments at 37°C for 48 h, and then stained with annexin V-FITC and propidium iodide (PI) to distinguish populations of early apoptotic (annexin V^+^/PI^−^), late apoptotic (annexin V^+^/PI^+^), and necrotic (annexin V^−^/PI^+^) using annexin V/PI Apoptosis Assay Kit (Sangon Biotech, Shanghai, China). The percentage of apoptotic cells was calculated as the sum of the percentages of early apoptotic and late apoptotic cells.

### Cell cycle analysis

MDA-MB-231 cells were incubated with various treatments at 37°C for 48 h. Following incubation, cells were fixed by the addition of absolute ethanol. After 24-h fixation, these samples were stained with PI (Beyotime Biotech, Shanghai, China). The proportion of singlet cells in G1, S and G2 were calculated with the MFLT32 analysis software.

### Immunoblotting assay

MDA-MB-231 cells were starved for 24 h in a 6-well plate, and then treated with series of concentrations of the treatments for 1 h, followed by stimulation with 10 ng/mL VEGF for another hour. We examined the effect of mAb04-MICA on tyrosine phosphorylation of VEGFR2 and downstream signal proteins AKT/ERK/P38 MAPK.

The expression of Bcl2/Bcl-xL/Bak/Bax was detected to identify the effect of mAb04-MICA on the apoptotic signaling pathways. Briefly, MDA-MB-231 cells were treated with series of concentrations of mAb04-MICA for 48 h after starvation. Protein preparation, quantification, and immunoblot analyses were performed as previously described [34]. All the antibodies were purchased from Cell Signaling Technology.

### RNA silencing of NKG2D on NK92 cells

NKG2D (GenBank accession number AJ001687.1) shRNA (5′- GGATCCCGGATGGGACTAGTACACATTCTTCAAGAGAGAATGTGTACTAGTCCCATCCTTTTTTCCAAGAATTC-3′) sequence was cloned into pLVX-shRNA vector (AXYBIO, Changsha, China). The plasmids were transfected into HEK293T cells using lipofectamine 2000 (Invitrogen, USA). Supernatant containing lentiviral plasmids was collected to infect NK92 cells. RNA silencing was analyzed using anti-NKG2D antibody by flow cytometry. The NKG2D silenced NK92 cells were used as effector cells in the cytotoxicity assay to verify if the fusion antibody will interfere the ADCC function of Fc.

### LDH release cytotoxicity assay

The CytoTox 96 Nonradioactive Cytotoxicity assay (Promega, Madison, USA) was performed based on the calorimetric detection of lactate dehydrogenase (LDH) released from the target cells. MDA-MB-231/MDA-MB-435 cells were co-cultured with various amounts of PBMCs or NK92-FcR cells in the presence or absence of the treatments for 4 h at 37°C. LDH analysis was performed following manufacturer's protocol. Controls for spontaneous LDH release in effector and target cells, as well as target maximum release, were prepared. The calculation of cytotoxicity percentage was as follows:

%Cytotoxicity=[(experimental-effector spontaneous-target spontaneous)/(target maximum−target spontaneous)]×100.

### Complement-dependent cytotoxicity

250 μL of MDA-MB-231 cells (1.2×10^6^ cells/mL) were harvested from culture, and the treatments or AK404R (scFv of mAb04 preserved in our lab) added to achieve the indicated final concentration. Triplicate samples were incubated for 15 min on ice. After incubation, rabbit serum (Sigma-Aldrich) was added as a source of complement to a final concentration of 10%, and samples were incubated at 37°C for 3 h. Cell viability was assessed by flow cytometry with PI staining.

### NK degranulation and cytokine production

NK92-FcR cells were incubated with IL-2 for 24 h and then co-cultured with MDA-MB-231/MDA-MB-435 cells with/without the presence of the treatments for 4 h at 10:1 E:T ratio. Degranulation of NK cells was evaluated on the basis of the expression of LAMP-1 (CD107a) as previously described [37, 38]. Meanwhile, at the end of the incubation, cells were permeabilized with Cytofix/Cytoperm (BD Biosciences) and further treated with anti-interferon-gamma (IFNγ)/tumor necrosis factor-alpha (TNF-α) antibody before analysis [37-40]. The anti-CD107a-FITC antibody, anti-IFNγ-PE/anti-TNF-α-PE antibody were purchased from Miltenyi Biotec. Secretory IFNγ and TNF-α levels were detected using commercial ELISA kits (KeyGEN Biotech, Nanjing, China), according to manufacturer's instructions. All indicated concentrations were expressed as means of triplicate measurements with SD.

### Xenograft model and administration

Breast cancer xenograft models were established by subcutaneously injecting MDA-MB-231/MDA-MB-435 cells (1×10^7^) into BALB/c nude mice (Yangzhou University Comparative Medicine Centre, Yangzhou, China). When the tumors reached 100 mm^3^, mice were randomized into different groups then treated with indicated treatments intravenously every 3 days. Tumor development was measured periodically and the tumor volume was determined using the formula V=(length×width^2^)/2. Mice were followed for survival and sacrificed when tumors reached 2000 mm^3^ as per institutional guidelines to determine the survival of tumor-bearing nude mice.

### Immunohistochemistry and immunofluorescence staining

Immunohistochemical (IHC) analysis was performed using antibodies against Ki-67, VEGF, CD31 (Cell Signaling Technology, USA) respectively. For immunofluorescence (IF) staining, sections were incubated with anti-CD56, anti-IFNγ, anti-TNF-α antibodies (Abcam, Cambridge, UK). Next, slides were mounted with Vectashield mounting media containing DAPI and were analyzed under fluorescence microscope.

### Statistical analysis

The data are indicated as means ± standard deviation (SD). Significance levels were estimated using the student's *t* test and P values of 0.05 or less were considered statistically significant. The calculation was performed with the GraphPad Prism software (San Diego, CA).

## SUPPLEMENTARY FIGURES



## Abbreviations

ADCC: antibody-dependent cellular cytotoxicity
ATCC: American type culture collection
CDC: complement dependent cytoxicity
CEA: carcino embryonie antigen
ECGS: endothelial cell growth supplement
ECM: endothelial culture medium
ER: estrogen receptor
Fab: antigen-binding fragments
FACS: fluorescence activated cell sorter
FBS: fetal bovine serum
Fc: fragment crystallizable
FcγRs: Fc gamma receptors
FITC: fluorescein isothiocyanate
HER2: human epidermal growth factor receptor-2
HUVECs: human umbilical vascular endothelial cells
IF: immunofluorescence
IFNγ: interferon-gamma
IHC: immunohistochemical
KDR3: the extracellular domain 3 of human VEGFR2
MHC: major histocompatibility complex
MICA/B: MHC class I-related chain molecules A and B
NKG2D: NK group 2, member D
OS: overall survival
PBMCs: peripheral blood mononuclear cells
PI: propidium iodide
PPE: palmar-plantar erythrodysesthesia
scFv: single chain fragment variable
SD: standard deviation
TAAs: tumor associated antigens
TNF-α: tumor necrosis factor-alpha
ULBPs: UL16-binding proteins
ULBP2: UL16-binding protein 2
VEGF: vascular endothelial growth factor
VEGFR2: vascular endothelial growth factor receptor 2.
