# Supplementary material for: VEGFR2 targeted antibody fused with MICA stimulates NKG2D mediated immunosurveillance and exhibits potent anti-tumor activity against breast cancer
Source: Oncotarget. 2016 Feb 19;7(13):16445–61. doi: 10.18632/oncotarget.7501 (PMC4941327; doi:10.18632/oncotarget.7501)
Supplement: Supplementary file 1 [file oncotarget-07-16445-s001.pdf]

SUPPLEMENTARY FIGURES

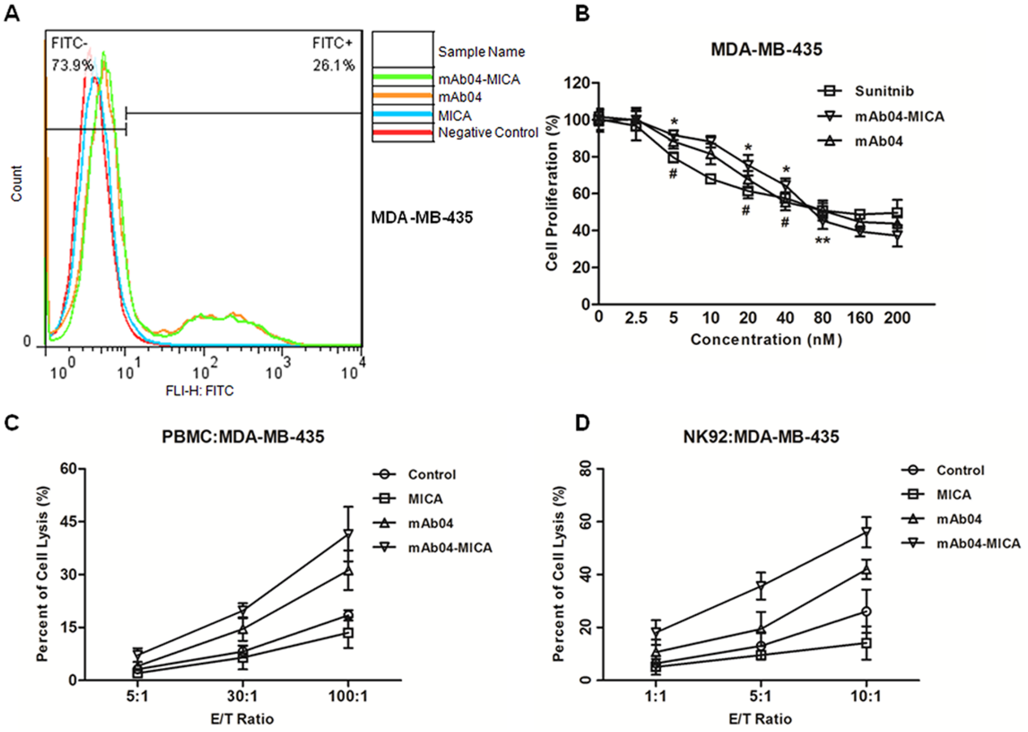

Supplementary Figure S1: mAb04-MICA inhibited the proliferation of MDA-MB-435 cells and enhanced PBMC/NK92 cell-mediated cytotoxicity. A. mAb04-MICA exhibited 26.1% binding rate with MDA-MB-435 cells. **B.** mAb04-MICA (Data were presented as the mean ± SD, n = 5, *p < 0.05, **p < 0.01)/mAb04 (n = 5, #p < 0.05, ##p < 0.01) inhibited the proliferation of MDA-MB-435 cells. **C, D.** MDA-MB-435 cells were used as target cells and PBMC/NK92 cells as effect cells for a LDH release assay. The increase of cell lysis was enhanced when treated with mAb04-MICA compared to mAb04. Measurements were performed in triplicate.

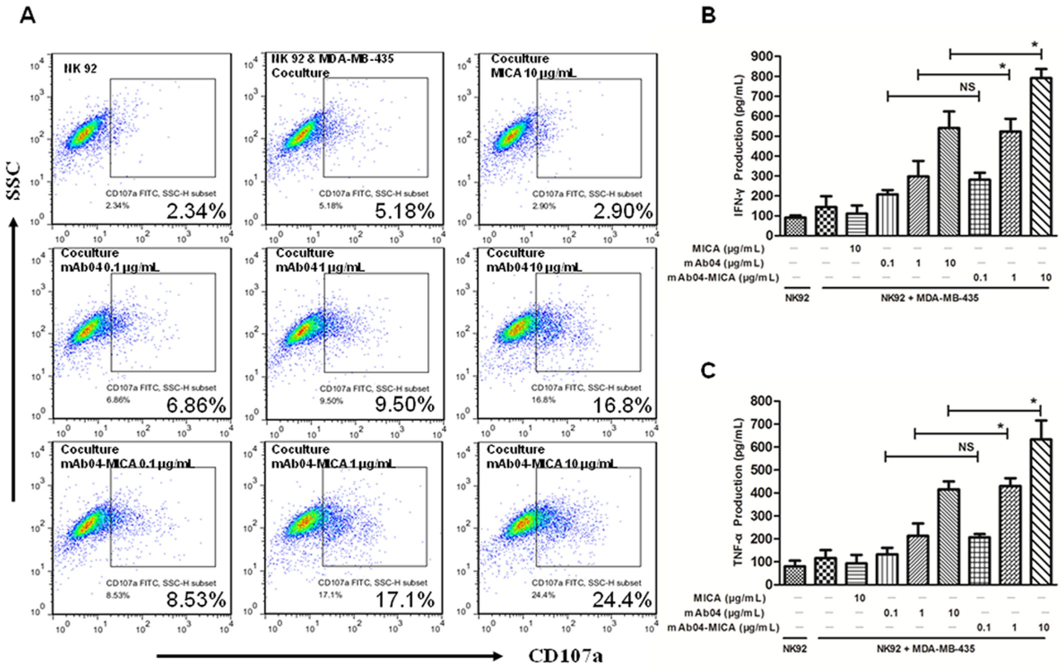

Supplementary Figure S2: Degranulation of NK92 and the expression of cytokines were increased in mAb04-MICA group compared to mAb04. A. Flow cytometry analysis of CD107a expression on NK92 cells after exposure to MDA-MB-435 cells for 4 h. The E/T ratio was 10:1. **B, C.** ELISA detected the IFN-γ and TNF-α concentrations after NK92 cells co-cultured with MDA-MB-231 cells for 4 h at E:T ratio (10:1). These results obtained on triplicate samples were presented as the mean ± SD, $^{*}p < 0.05$, $^{**}p < 0.01$.

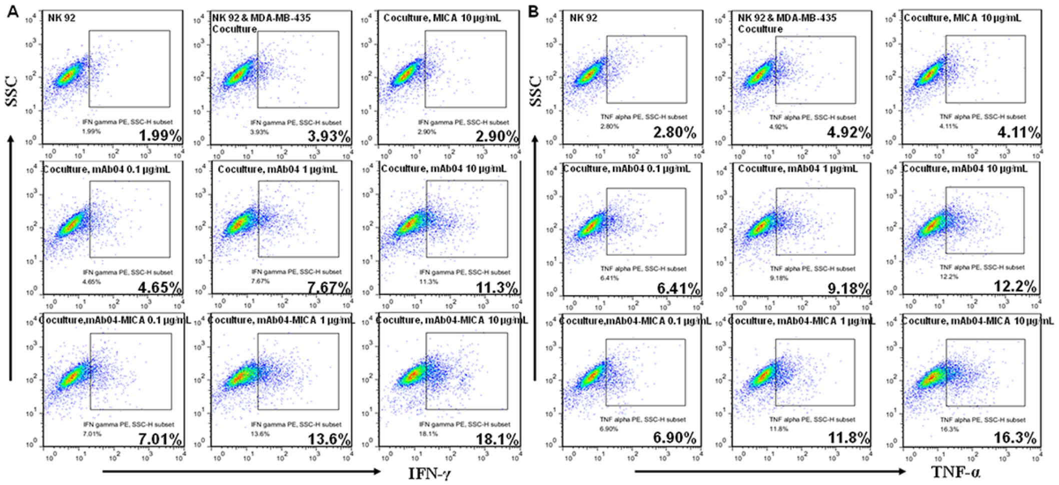

Supplementary Figure S3: NK92 cells secreted more cytokines when treated with mAb04-MICA in the coculture with MDA-MB-435 cells. A, B. Flow cytometry data represented the distribution of cytokine positive cells among NK92 cells, which indicated the proportion of NK92 cells expressing IFN-γ/TNF-α along the x-axis increased when concentration of the treatments increased. The percentage of IFN-γ/TNF-α positive cells was calculated by FlowJo software.

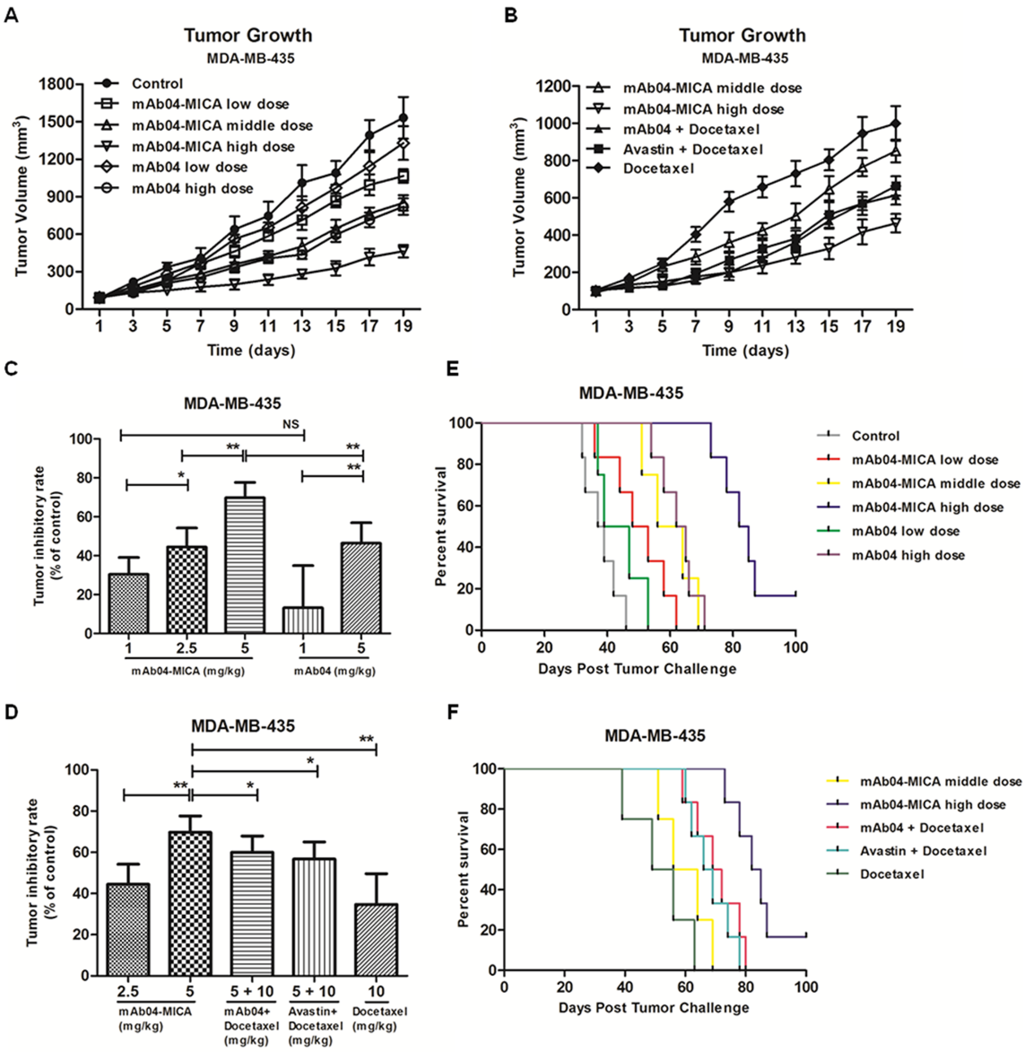

Supplementary Figure S4: mAb04-MICA demonstrated *in vivo* efficacy against a MDA-MB-435 xenograft. A, B. Tumor growth curves for nude mice. Each nude mice was subcutaneously injected 1×10^7 MDA-MB-435 cells. Treatment began when the average tumor volume reached 100 mm^3. **C, D.** Tumor inhibition rates of different dosage groups. mAb04-MICA significantly improved tumor inhibition rate compared to mAb04/Docetaxel. Data were presented as the mean ± SD, $*p < 0.05$, $**p < 0.01$. **E, F.** Survival curves for nude mice bearing tumor. mAb04-MICA had noteworthy survival benefit compared to mAb04 + Docetaxel or Avastin + Docetaxel.

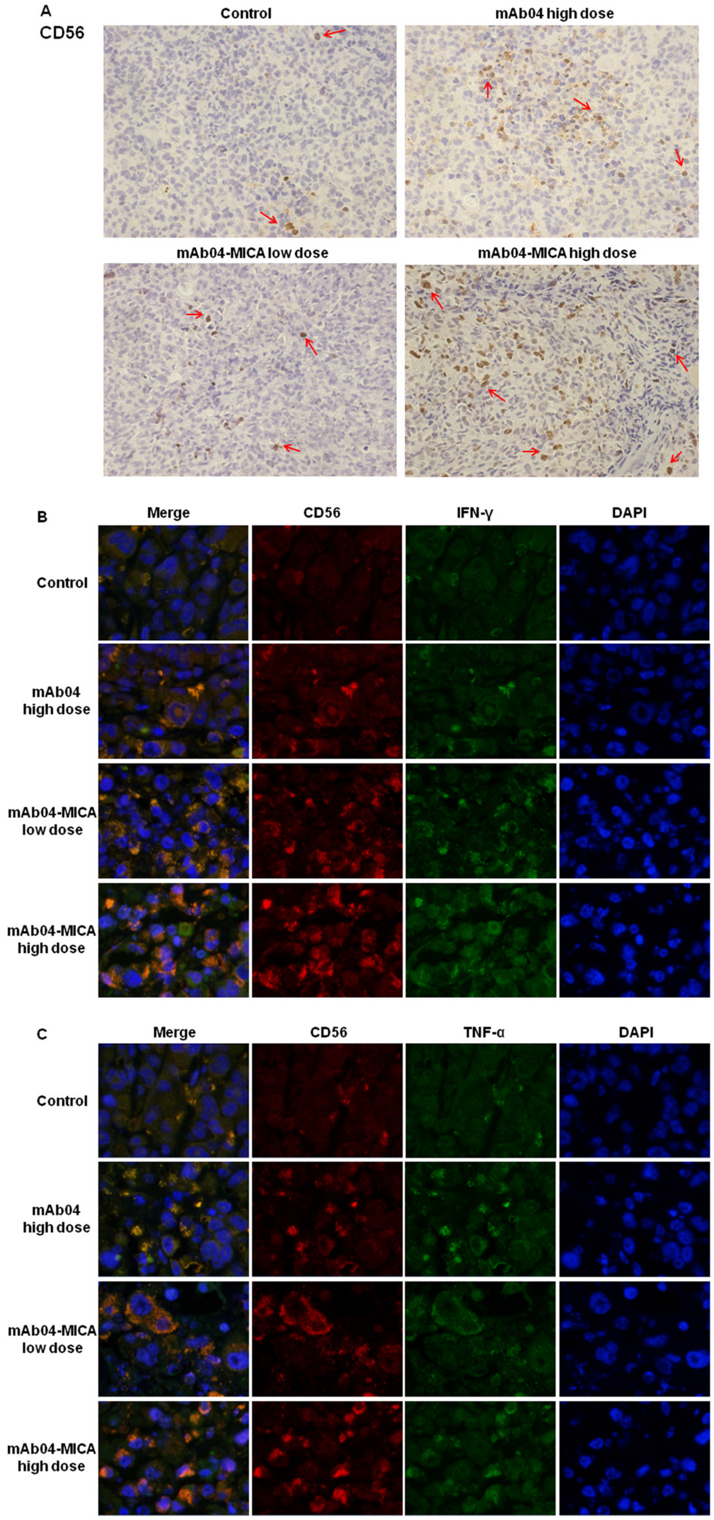

Supplementary Figure S5: mAb04-MICA increased the infiltration of NK cells in MDA-MB-435 tumor tissue and increased the production of IFN-γ and TNF-α by NK cells. A. $CD56^{+}$ cells were detected by IHC staining (brown staining, indicated by the red arrows) on serial sections, demonstrating more distribution of NK cells with mAb04-MICA treatment. **B, C.** IF double staining of CD56 (red fluorescence) and IFN-γ/TNF-α (green fluorescence) to determine the expression level of IFN-γ/TNF-α by NK cells. The orange staining cells after merged indicated the IFN-γ/TNF-α expressing NK cells, which increased with the dose.

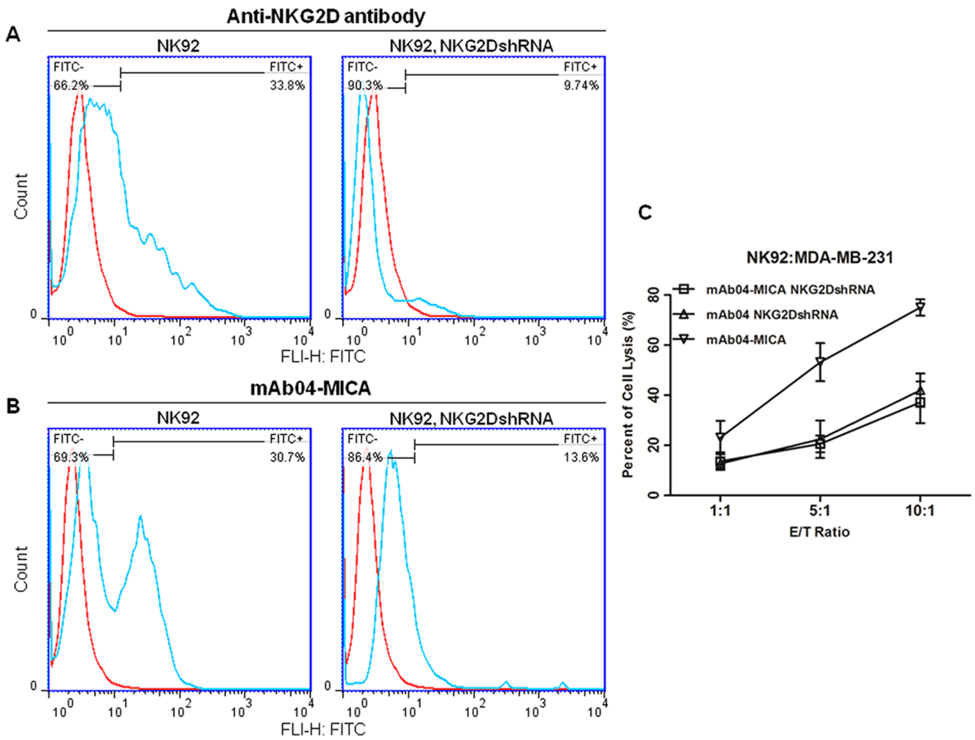

Supplementary Figure S6: The fusion antibody mAb04-MICA would not interfere the ADCC function of Fc. A. The binding rate of anti-NKG2D antibody to NK92 cells decreased from 33.8% to 9.74% after NKG2D targeted RNA silencing. **B.** The binding rate of mAb04-MICA to NK92 cells decreased from 30.7% to 13.6% after NKG2D targeted RNA silencing. **C.** The cytotoxicity reduced obviously when RNA silencing NKG2D on NK92 cells, but maintained similarity to that of mAb04.

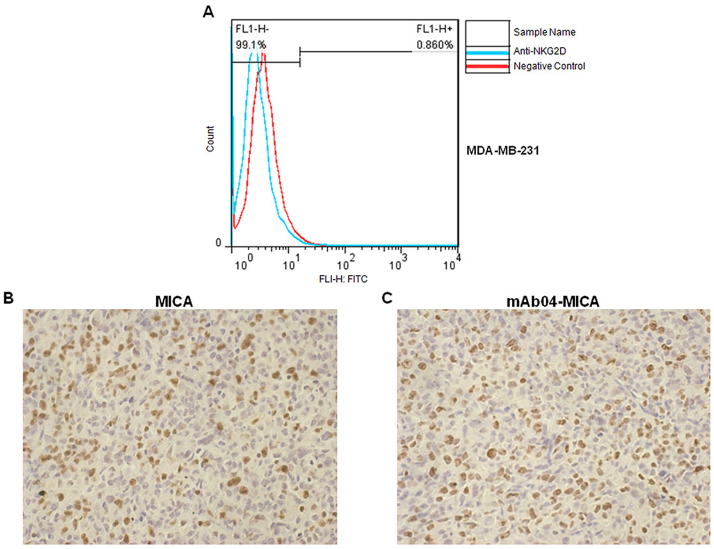

Supplementary Figure S7: High specific binding rate to mouse NK cells of MICA and mAb04-MICA. A. Flow cytometry showed there was little NKG2D expressed on MDA-MB-231 cells. **B.** Figure 11A demonstrated the sections from mAb04-MICA (5 mg/kg) treated group were obviously infiltrated by NK cells. The sections were layered with MICA and followed by incubation with a labeled anti-human MICA antibody as positive control. **C.** Sections were blocked by mAb04, subsequently layered with mAb04-MICA, followed by incubation with a labeled anti-human MICA antibody. The brown staining in B and C indicated NK cells, and revealed MICA and mAb04-MICA could bind to mouse NK cells.
